# β-Aminobutyric acid enhances *Arabidopsis thaliana* resistance to the hemibiotrophic fungal pathogen *Colletotrichum higginsianum*

**DOI:** 10.5511/plantbiotechnology.26.0119a

**Published:** 2026-06-25

**Authors:** Mari Narusaka, Yoshihiro Narusaka

**Affiliations:** 1 Okayama Prefectural Technology Center for Agriculture, Forestry, and Fisheries, Research Institute for Biological Sciences, Okayama 716-1241, Japan

**Keywords:** *Arabidopsis thaliana*, β-aminobutyric acid, *Colletotrichum higginsianum*, induced resistance, salicylic acid

## Abstract

β-Aminobutyric acid (BABA) is a nonproteinogenic amino acid that functions as a potent plant defense activator, conferring broad-spectrum resistance in many plants. Although its effects on biotrophic and necrotrophic pathogens are well known, the molecular basis of BABA-induced resistance (BABA-IR) against the hemibiotrophic fungus *Colletotrichum higginsianum* remains unclear. In this study, we investigated BABA’s effects on *C. higginsianum*-infected *Arabidopsis thaliana*. Foliar BABA application (1.25–10 mM) reduced lesion size and fungal biomass in a concentration-dependent manner, with 5–10 mM being most effective. Microscopy showed that BABA treatment prevented fungal invasion beyond the appressorium stage. Mutant analysis revealed that BABA-IR depends on salicylic acid (SA) biosynthesis (*NahG* and *eds16-1*) and the signaling components EDS1 and NDR1. In contrast, mutants insensitive to jasmonic acid and ethylene (*jar1-1* and *ein2-12*, respectively) retained BABA-mediated protection. These findings indicate that BABA primes SA-dependent defenses against *C. higginsianum* while highlighting its potential for improving sustainable crop protection.

In nature, plants encounter diverse microbial pathogens, including fungi, bacteria, and viruses, although only a few cause severe damage. Plants possess sophisticated surveillance systems that recognize such pathogens and rapidly activate defenses. Systemic acquired resistance (SAR) is an induced response in uninfected plant organs after inoculation with microorganisms. SAR, which provides long-lasting immunity against a broad spectrum of pathogens, is activated when levels of the endogenous defense hormone salicylic acid (SA) become elevated and induce expression of SA-responsive genes (Ryals et al. 1996).

Several natural and synthetic chemicals, known as plant defense activators or plant activators, induce SAR-like responses and expression of defense-related genes, including pathogenesis-related genes. These chemicals and pathogens trigger the same resistance spectrum and SAR-associated gene activation (Lawton et al. 1996; Uknes et al. 1992). Notably, 2,6-dichloroisonicotinic acid, benzo(1,2,3)thiadiazole-7-carbothioic acid *S*-methyl ester, *N*-cyanomethyl-2-chloroisonicotinamide, probenazole, imprimatins, pyrimidine-type plant activators, and thienopyrimidine-type plant activators have all been reported to induce resistance in plants (Friedrich et al. 1996; Lawton et al. 1996; Nakashita et al. 2002; Narusaka and Narusaka 2017; Narusaka et al. 1999; Noutoshi et al. 2012; Sun et al. 2015; Yasuda et al. 2003; Yoshioka et al. 2001).

β-Aminobutyric acid (BABA), a nonproteinogenic amino acid, is a potent plant defense activator that enhances plant resistance against a broad spectrum of organisms, including viruses, bacteria, oomycetes, fungi, nematodes, and aphids (Cohen 2002; Cohen et al. 2016; Hodge et al. 2006; Jakab et al. 2001; Zimmerli et al. 2000, 2001). However, BABA has no direct antimicrobial effect on pathogens (Kim and Kang 2023; Zimmerli et al. 2000). In *Arabidopsis thaliana*, BABA-induced priming of SA-dependent defenses protects against *Pseudomonas syringae* and *Botrytis cinerea*, whereas BABA-mediated callose-rich papillae formation and early penetration resistance at the cell wall help protect against *Hyaloperonospora parasitica*, *Plectosphaerella cucumerina*, *Alternaria brassicicola*, and *Hyaloperonospora arabidopsidis* (Tao and Ton 2024; Ton and Mauch-Mani 2004; Zimmerli et al. 2000, 2001).

*Colletotrichum higginsianum* causes anthracnose lesions on leaves, petioles, and stems in turnip, mustard, and Chinese cabbage, leading to serious yield losses (Higgins 1917; Narusaka et al. 2004). Inoculation of *A. thaliana* accession Columbia (Col-0) leaves with *C. higginsianum* results in fungal growth and lesions similar to those observed in other crucifers inoculated under the same conditions (Narusaka et al. 2004). *Colletotrichum higginsianum* is a hemibiotroph, initially biotrophic in early infection stages and later necrotrophic. Its ability to cause anthracnose disease in *A. thaliana* makes it an attractive pathosystem for studying fungal pathogenicity and plant defenses.

In the present study, we analyzed the effects of BABA on infection by *C. higginsianum*. Although BABA-induced resistance (BABA-IR) against *C. higginsianum* was previously demonstrated in *Brassica rapa* (Kim et al. 2013), its molecular mechanisms remain poorly understood. We employed *A. thaliana* wild-type and signaling mutants to explore the SA, jasmonic acid (JA), and ethylene (ET) pathways’ contributions to BABA-primed resistance. We found that BABA-treated *A. thaliana* Col-0 showed activation of the SA pathway, which conferred resistance to *C. higginsianum*. Additionally, BABA treatment protected JA- and ET-insensitive mutants but was less effective in plants with impaired SA signaling, including *NahG* transgenic plants and *eds16-1*, *eds1-1*, *ndr1-1*, and *npr1-1* mutants. These findings demonstrate that the SA-dependent signaling pathway plays a central role in BABA-IR against *C. higginsianum*.

*Colletotricum higginsianum* (MAFF305635) isolate was obtained from the Ministry of Agriculture, Forestry and Fisheries Genebank, Japan. Culture was maintained in darkness on potato dextrose agar (PDA; Difco, Detroit, MI, USA) at 24°C. Conidia were collected by gently scraping cultures incubated for 7–10 days and filtering through two layers of sterile cheesecloth (Narusaka et al. 2004).

*Arabidopsis thaliana* plants were grown for 28–30 days in a growth chamber at 22°C under a 12/12-h light/dark cycle. Potting soil comprised a mixture of Supermix A (Sakata Seed Corp., Yokohama, Japan), expanded vermiculite (1.5–2.0 mm granules; Asahikogyo Corp., Okayama, Japan), and pearlite (1.5–3.0 mm granules; Taiheiyo Materials Corp., Tokyo, Japan) in a 2 : 1 : 1 ratio. Plants were inoculated by spraying leaves with a conidial suspension of *C. higginsianum* (5×10^5^ conidia ml^−1^ in distilled water [DW]). Inoculated plants were maintained in a growth chamber at 22°C with 100% relative humidity under a 12/12-h light/dark cycle. Control plants were treated with DW only. Plants were harvested at 5 days postinoculation (dpi) for quantitative reverse transcription polymerase chain reaction (qRT-PCR) analysis. Using TRIZOL reagent according to the manufacturer’s protocol (Thermo Fisher Scientific Inc., Waltham, MA, USA), total RNA was isolated from leaves flash-frozen in liquid nitrogen immediately after harvesting. *Colletotrichum higginsianum* was quantified using the method described by Narusaka et al. (2010). Fungal hyphae in lesions were visualized by staining inoculated leaves with lactophenol–trypan blue (Narusaka et al. 2003).

Regarding BABA’s toxic effects on *C. higginsianum*, Kim et al. (2013) reported that it lacked in vitro antifungal activity against isolate C97-28 (KACC 40807). We also tested isolate MAFF305635 on PDA with or without 5 mM BABA. BABA was added to the PDA medium after autoclaving when the temperature had cooled to below 50°C. For colony growth measurements, eight PDA plates were prepared and a mycelial plug was placed at the center of each plate. The plates were incubated at 24°C in darkness, and the colony diameter was measured. As a result, *C. higginsianum* grew similarly on both media (Table 1).

**Table 1:** Table 1. Colonies of *Colletotricum higginsianum* formed on PDA media supplemented with 5 mM BABA.

	Treatment	
	DW	5 mM BABA
Diameter (mm)*	50.6±0.61	52.25±1.01

*Colony diameter was measured 7 days after culture under dark conditions at 24°C. No statistically significant difference between treatments.

BABA is known to protect *Arabidopsis* against *Peronospora parasitica* and *P. syringae* pv. *tomato* DC 3000 (Zimmerli et al. 2000), pathogens that activate SA-dependent signaling. Conversely, it enhances *Arabidopsis* resistance to the necrotrophic fungal pathogen *B. cinerea* through JA/ET signaling (Zimmerli et al. 2001). In the present study, we extended analysis to a hemibiotrophic pathogen that induces defense responses via SA and/or JA/ET signaling.

The concentration of BABA required for effective resistance under controlled conditions depends on the host, pathogen, and application method (Cohen 2002). *Arabidopsis thaliana* Col-0 plants were pretreated with 1.25–10 mM BABA via foliar spray, followed by inoculation with *C. higginsianum* 2 day later. Treatment with 1.25 or 2.5 mM BABA moderately reduced disease symptoms and pathogen load, whereas higher concentrations (5–10 mM) visibly decreased the extent of necrosis at 5 dpi. Treatments with 5 and 10 mM BABA effectively protected *A. thaliana* leaves against anthracnose compared with the control, as supported by quantitative analysis of fungal biomass (Figure 1A). Microscopy revealed that 5 mM BABA blocked fungal penetration and invasion after appressorium formation (Figure 1C, D, E, F). In contrast, soil drench with 5 mM BABA failed to protect Col-0 plants against the pathogen (Figure 1B). The difference between foliar and soil drench applications may be attributed to the mode of BABA uptake. Foliar spraying allows direct absorption through leaf tissues, whereas soil application may restrict uptake via roots or result in partial adsorption or degradation in the soil.

**Figure 1:**
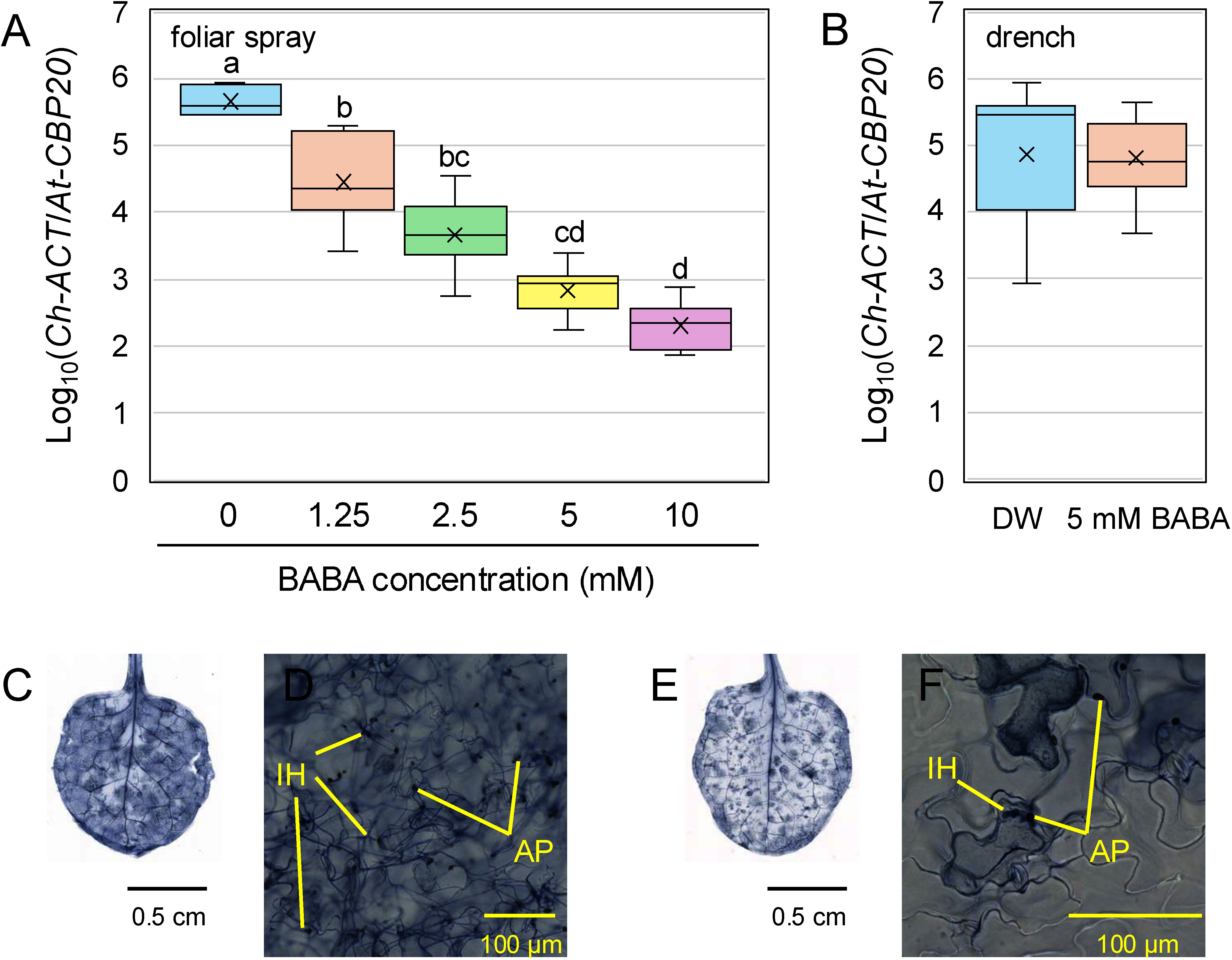
Figure 1. Effect of BABA on *C. higginsianum* infection in *Arabidopsis thaliana* Col-0. Plants grown for 28–30 days (12/12-h light/dark cycle) were pretreated with 1.25–10 mM BABA via foliar sprays (A, C–F) or 5 mM BABA via soil drench (B), followed by inoculation with *C. higginsianum* (5×10^5^ conidia ml^−1^ DW) 2 days later. (A, B) Six inoculated leaves were harvested at 5 dpi, and total RNA was isolated. Subsequently, qRT-PCR was performed with *Ch-ACT* and *At-CBP20* primer pairs to quantify *Ch-ACT* mRNA expression. Experiments (A, B) were conducted independently at least twice (*n*>3 per experiment), and data were combined. (A) Different letters indicate significant differences between control and treated plants (Tukey–Kramer multiple comparison test, *p*<0.01). (B) There were no significant differences between DW- and BABA-treated plants (Dunnett’s multiple comparison test, *p*<0.01). (C–F) Fully expanded leaves were selected for microscopic observation because infection severity varies depending on leaf age. Light micrographs of leaf surfaces after clearing and trypan blue staining at 5 dpi. Images of control (DW-treated) plants (C, D) and 5 mM BABA-treated plants (E, F) were acquired using an all-in-one fluorescence microscope (BZ-X800, KEYENCE, Osaka, Japan). Image (F) was acquired as a Z-stack using the BZ-X800 system and processed into a Z-projection via the 3D application (BZ-H4R) in BZ-X Analyzer software (BZ-H4A, both KEYENCE). AP, appressorium; IH, infection hypha. Each image (C–F) represents three independent experiments.

BABA’s mode of action was tested using transgenic *Arabidopsis* and mutants impaired in their signal-transduction pathways. Plants were sprayed with 5 mM BABA and inoculated with *C. higginsianum* 2 days later. Samples were collected at 5 dpi for qRT-PCR analysis. *NahG* and *eds16-1* are SA-deficient *Arabidopsis* genotypes: *NahG* expresses a chimeric transcription unit encoding an SA hydroxylase (Gaffney et al. 1993), and *eds16-1* carries a point mutation in the SA biosynthetic enzyme isochorismate synthase 1 (Wildermuth et al. 2001). The *npr1-1* mutant is impaired in SA signaling: *npr1-1* has a point mutation in an IκB-like downstream signal-transduction component (Cao et al. 1997). All mutants are nonresponsive to SAR inducers (Cao et al. 1994). Treatment with 5 mM BABA moderately reduced disease incidence in *npr1-1* but did not enhance resistance against *C. higginsianum* in *NahG* or *eds16-1* (Figure 2). EDS1 and NDR1 function as key regulators that coordinate SA signaling and race-specific disease resistance. In addition to their role in SA-dependent defenses, *EDS1* and *NDR1* also encode essential components of race-specific disease resistance (Aarts et al. 1998; Century et al. 1995; Falk et al. 1999). BABA did not protect *eds1-1* and *ndr1-1* mutants against *C. higginsianum* (Figure 2, 3). Differences between DW- and BABA-treated plants were evaluated separately for each genotype using Welch’s *t*-test with Bonferroni correction. No statistically significant differences were detected in either the Ws-2 or *eds1-1* background, indicating that BABA-induced resistance is not evident in these genotypes under our experimental conditions.

**Figure 2:**
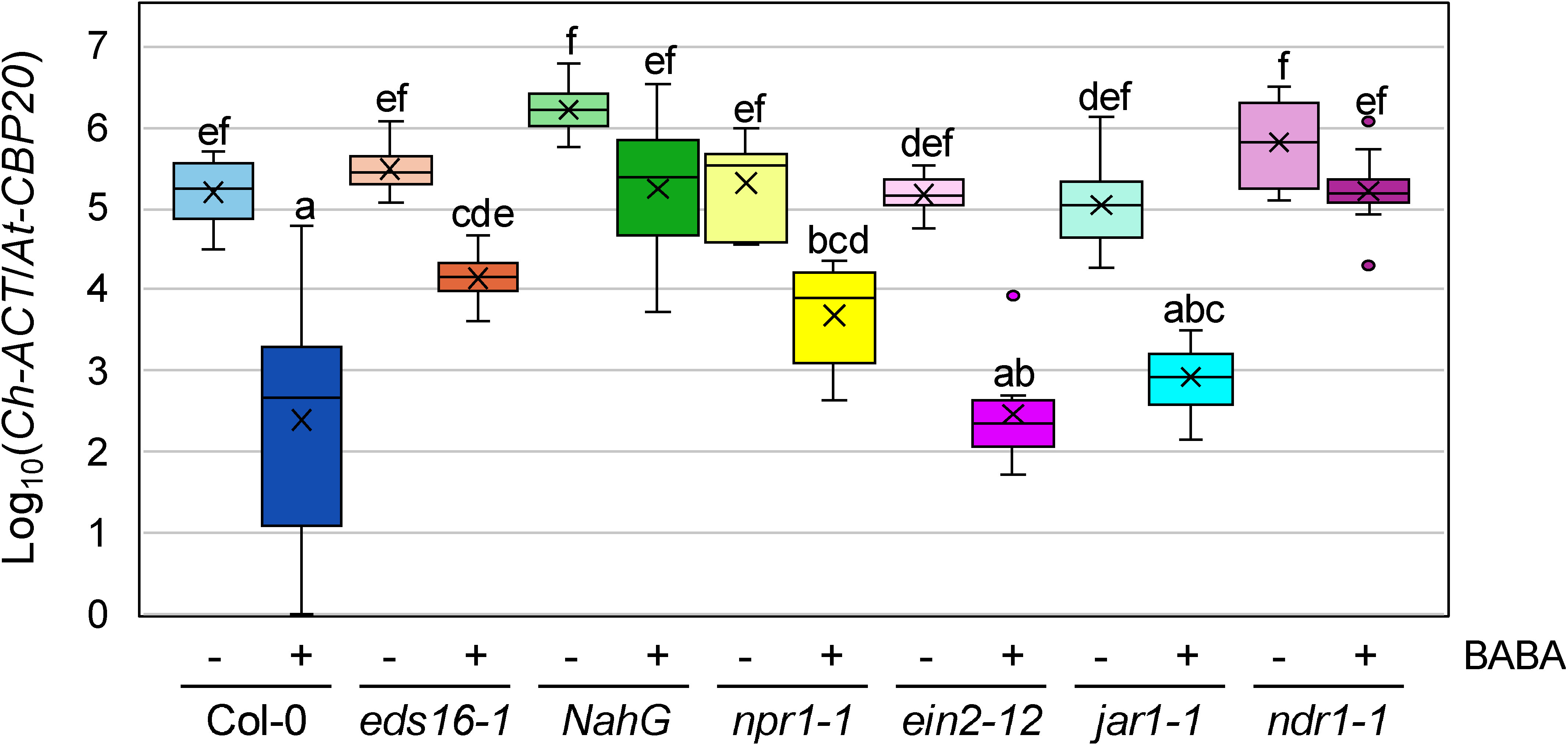
Figure 2. Quantification of *C. higginsianum* in inoculated transgenic *Arabidopsis* or signaling mutants (Col-0 background) by qRT-PCR. Plants grown for 28–30 days (12/12-h light/dark cycle) were pretreated with distilled water (DW) or 5 mM BABA via foliar sprays, followed by inoculation with *C. higginsianum* (5×10^5^ conidia ml^−1^ DW) 2 day later. Six inoculated leaves were harvested at 5 dpi, and total RNA was isolated. Quantification of fungal growth was performed by qRT-PCR using primer pairs specific for *Ch*-*ACT* and *At*-*CBP20*. Data represent combined results from at least two independent experiments (*n*>3 per experiment). For each genotype, DW- and BABA-treated samples are shown side-by-side to allow direct comparison. Different letters indicate significant differences among treatments (Tukey–Kramer multiple comparison test, *p*<0.01).

**Figure 3:**
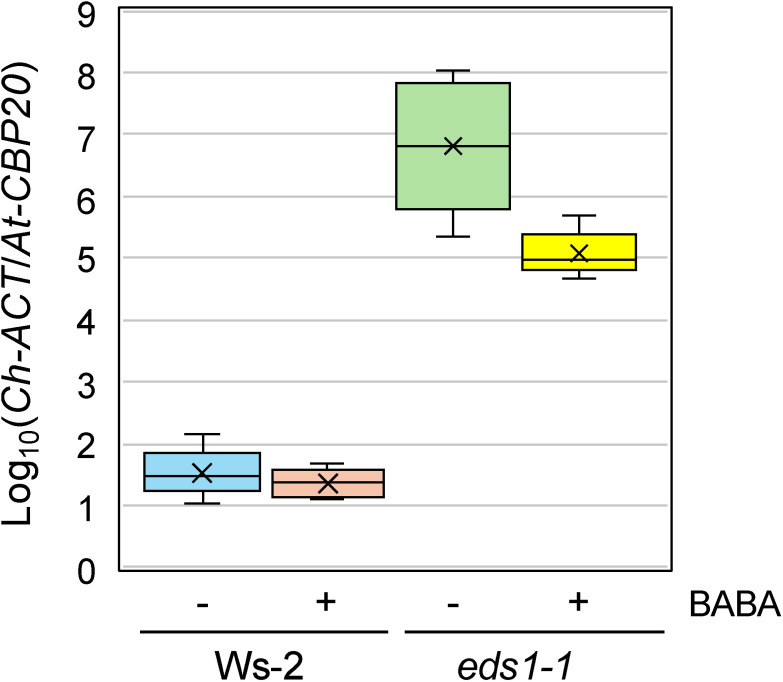
Figure 3. Quantification of *C. higginsianum* in inoculated *Arabidopsis* signaling mutant (Ws-2 background) by qRT-PCR. Plants grown for 28–30 days (12/12-h light/dark cycle) were pretreated with distilled water (DW) or 5 mM BABA via foliar sprays, followed by inoculation with *C. higginsianum* (5×10^5^ conidia ml^−1^ DW) 2 days later. Six inoculated leaves were harvested at 5 dpi, and total RNA was isolated. Subsequently, qRT-PCR was performed with *Ch-ACT* and *At-CBP20* primer pairs to quantify *Ch-ACT* mRNA expression. Experiments were conducted independently at least twice (*n*>3 per experiment), and data were combined. For each genotype, differences between DW- and BABA-treated plants were evaluated using Welch’s *t*-test with Bonferroni correction. No statistically significant differences were detected between DW- and BABA-treated plants in the Ws-2 background (*p*≥0.025 after correction).

The contribution of JA/ET signaling to BABA-mediated protection against *C. higginsianum* in *Arabidopsis* was assessed using ET-insensitive *ein2-12* and JA-deficient *jar1-1*. BABA treatment reduced fungal growth in *ein2-12* at 5 dpi and protected *jar1-1* mutants (Figure 2).

The hemibiotrophic fungus *C. higginsianum* has two infection strategies: an initial biotrophic phase followed by a necrotrophic phase (Narusaka et al. 2004). Thus, hosts may require distinct defenses during these transitions. Previous studies suggested that *A. thaliana* activates an SA-dependent signaling response early in *C. higginsianum* infection, followed by JA-dependent defenses (Narusaka et al. 2004). Moreover, gene expression profiling showed that BABA-treated Col-0 plants exhibited stress-related patterns more similar to those induced by SA than by JA or ET (Narusaka et al. 2006).

In the present study, we showed that BABA protects *Arabidopsis* against *C. higginsianum*. Foliar BABA application inhibited fungal infection, and this protection was not due to direct antifungal activity. Instead, BABA appeared to function as a priming agent that activates plant immunity. Moreover, BABA likely stimulated the SA-dependent pathway downstream of SA biosynthesis during early infection.

BABA treatment failed to fully protect SA-deficient *NahG* plants and the SA biosynthesis mutant *eds16-1* (*EDS16*/*SID2*), indicating that SA biosynthesis is essential for BABA-IR. Although our results do not directly show the absence of antifungal activity, the persistence of fungal infection in these mutants suggests that BABA does not act through direct inhibition of *C. higginsianum*. In contrast, the *npr1-1* mutant exhibited moderate protection, suggesting that NPR1 is nonessential for BABA-IR. Additionally, BABA-treated JA- and ET-insensitive mutants (*jar1-1* and *ein2-12*, respectively) displayed protection levels similar to those of BABA-treated wild-type Col-0 plants, further supporting the SA-dependent nature of BABA-IR.

Our findings also indicate that EDS1 and NDR1 are required for BABA-IR. The inability of *ndr1-1* and *eds1-1* mutants to develop resistance after BABA treatment suggests that these components are essential not only for resistance gene–mediated resistance but also for BABA-induced defenses. EDS1 and NDR1 have also been implicated in harpin-induced resistance, with harpin activating distinct pathways mediated by various resistance proteins and elicitors (Dangl and Jones 2001; Dong 2001; Peng et al. 2003). However, the precise mechanism underlying the coordination of EDS1 and NDR1 activity in BABA-IR remains unknown.

Because *eds1-1* is in the Wassilewskija (Ws-2) background, whereas the other mutants used in this study are in the Col-0 background, direct comparisons should be interpreted with caution, as ecotype-dependent differences in disease responses may influence the observed effects. Furthermore, the Ws-2 ecotype exhibits relatively high basal resistance to *C. higginsianum* (Narusaka et al. 2009), which may limit the detection of additional protective effects conferred by BABA under our experimental conditions. Therefore, although our results support a role for EDS1 in BABA-induced resistance, we refrain from drawing definitive conclusions regarding its requirement in the Ws-2 background.

In summary, BABA reduces anthracnose severity on *A. thaliana* leaves and effectively primes resistance against *C. higginsianum*. Overall, our results highlight the potential of BABA as a plant defense activator for disease management.

## Abbreviations

BABA: β-aminobutyric acid
DW: distilled water
ET: ethylene
JA: jasmonic acid
PDA: potato dextrose agar
SA: salicylic acid
SAR: systemic acquired resistance
